# Sofosbuvir Suppresses the Genome Replication of DENV1 in Human Hepatic Huh7 Cells

**DOI:** 10.3390/ijms25042022

**Published:** 2024-02-07

**Authors:** Madoka Kurosawa, Fumihiro Kato, Takayuki Hishiki, Saori Ito, Hiroki Fujisawa, Tatsuo Yamaguchi, Misato Moriguchi, Kohei Hosokawa, Tadashi Watanabe, Noriko Saito-Tarashima, Noriaki Minakawa, Masahiro Fujimuro

**Affiliations:** 1 Department of Cell Biology, Kyoto Pharmaceutical University, Kyoto 607-8412, Japan; white.mint.leaf@gmail.com (M.K.); itous@belle.shiga-med.ac.jp (S.I.); hi-fujisawa@kenei-pharm.com (H.F.); yomiyama1998@nibiohn.go.jp (T.Y.); cat-060530@ezweb.ne.jp (M.M.); derumo.jazz@gmail.com (K.H.); 2 Department of Virology III, National Institute of Infectious Diseases, Tokyo 208-0011, Japan; fumihiro@niid.go.jp; 3 Research Center for Drug and Vaccine Development, National Institute of Infectious Diseases, Tokyo 162-8640, Japan; hishiki@niid.go.jp; 4 Department of Virology, Graduate School of Medicine, University of the Ryukyus, Okinawa 903-0215, Japan; twatanab@med.u-ryukyu.ac.jp; 5 Graduate School of Pharmaceutical Science, Tokushima University, Tokushima 770-8505, Japan; noriko.tarashima@tokushima-u.ac.jp (N.S.-T.); minakawa@tokushima-u.ac.jp (N.M.)

**Keywords:** sofosbuvir, PS-7977, (2′R)-2′-deoxy-2′-fluoro-2′-methyluridine, PSI-6206, GS-331007, dengue virus, DENV1, antiviral drug, replicon, RNA-dependent RNA polymerase

## Abstract

Dengue virus (DENV) causes dengue fever and dengue hemorrhagic fever, and DENV infection kills 20,000 people annually worldwide. Therefore, the development of anti-DENV drugs is urgently needed. Sofosbuvir (SOF) is an effective drug for HCV-related diseases, and its triphosphorylated metabolite inhibits viral RNA synthesis by the RNA-dependent RNA polymerase (RdRp) of HCV. (2′R)-2′-Deoxy-2′-fluoro-2′-methyluridine (FMeU) is the dephosphorylated metabolite produced from SOF. The effects of SOF and FMeU on DENV1 replication were analyzed using two DENV1 replicon-based methods that we previously established. First, a replicon-harboring cell assay showed that DENV1 replicon replication in human hepatic Huh7 cells was decreased by SOF but not by FMeU. Second, a transient replicon assay showed that DENV1 replicon replication in Huh7 cells was decreased by SOF; however, in hamster kidney BHK-21 cells, it was not suppressed by SOF. Additionally, the replicon replication in Huh7 and BHK-21 cells was not affected by FMeU. Moreover, we assessed the effects of SOF on infectious DENV1 production. SOF suppressed infectious DENV1 production in Huh7 cells but not in monkey kidney Vero cells. To examine the substrate recognition of the HCV and DENV1 RdRps, the complex conformation of SOF-containing DENV1 RdRp or HCV RdRp was predicted using AlphaFold 2. These results indicate that SOF may be used as a treatment for DENV1 infection.

## 1. Introduction

Recently, many emerging and re-emerging infectious diseases caused by life-threatening RNA viruses have been reported worldwide, including SARS-CoV2, the Ebola virus, the Zika virus, and the dengue virus (DENV). DENV, a single positive (+)-stranded RNA virus of the family *Flaviviridae*, is classified into four serotypes (i.e., DENV1-4) and is transmitted among humans through bloodsucking by *Aedes* mosquitoes [1,2]. DENV is known to cause dengue fever, dengue hemorrhagic fever, and dengue shock syndrome [3]. Dengue fever presents relatively mild clinical symptoms; however, dengue hemorrhagic fever leads to the life-threatening dengue shock syndrome. The mortality rates of dengue hemorrhagic fever in untreated cases exceed 20% but decrease to less than 1% with proper medical care [4,5]. Since 390 million people are infected annually with DENV, resulting in 20,000 deaths/year in tropical and subtropical countries [4,5,6], drugs targeting DENV are urgently needed.

In DENV infection, after virus entry into host cells, the DENV RNA genome is translated to a single polypeptide which contains structural proteins (C, E, and preM) and viral enzymes (NS1, NS2A/B, NS3, NS4A/B, and NS5) [7,8]. NS5, the RNA-dependent RNA polymerase (RdRp) of DENV, is thought to be an attractive drug target because of its pivotal role in the replication of the DENV RNA genome [9]. During viral replication, NS5 forms a viral RNA replicase complex with NS2A/B, NS3, and NS4A/B in the endoplasmic reticulum (ER). A number of nucleosides and their prodrugs have been developed as inhibitors against the RdRps of several RNA viruses [10,11,12,13], including the DENV RdRp [9,14,15,16,17]. These nucleoside analogs require mono-, di- and triphosphorylation by cellular nucleoside and nucleotide kinases to become the active 5′-triphosphorylated form. Some triphosphorylated nucleoside analogs can bind to the active site of the RdRp and compete with the natural nucleotide substrates for incorporation into RNA, which is followed by chain termination of viral RNA synthesis [16,17]. The development of the nucleotide type RdRp inhibitor, SOF, was a breakthrough for the treatment of HCV and provided a ray of hope to many patients with HCV-related disease [10,11,18,19,20]. In contrast, currently, no anti-DENV drugs have been approved for the clinical treatment of DENV infection/disease. 

The repurposing of a marketed antiviral drug for other viral infections is a good strategy to quickly address insufficient medical needs because the safety profile and virus-specific effects have already been demonstrated. As for drug targets directed against many RNA viruses, the viral RdRp has been thought to be a valid drug target. Sofosbuvir (also referred to as SOF, PS-7977, or GS-7977) is an effective drug for HCV-related diseases, and its phosphorylated metabolite is an inhibitor of the HCV RdRp (NS5B) [10,11,18,19,20]. (2′R)-2′-Deoxy-2′-fluoro-2′-methyluridine (FMeU, PSI-6206, RO2433, or GS-331007) and FMeU-triphosphate (FMeU-TP) are the ultimate metabolites produced from SOF in the human hepatocyte. FMeU-TP, which is the 5’-triphosphate metabolite derived from SOF, inhibits HCV RdRp-mediated viral RNA replication. Meanwhile, FMeU, which is the nucleoside metabolite derived from SOF (i.e., ProTide-detached SOF), has no inhibiting effect on HCV RdRp [21,22]. In the human hepatic cells, SOF is thought to be converted into an intermediate metabolite, FMeU-MP (GS-606965), by the detachment of iso-propanol, phenol, and alanine [21,22]. Finally, FMeU-MP is metabolized into either the phosphorylated active form (FMeU-TP) or dephosphorylated form (FMeU). Because FMeU is a poor substrate for cellular nucleoside kinases, FMeU is thought to be not converted into the triphosphate form [23]. However, FMeA is the most abundant metabolite detected in the blood of SOF-treated patients [24]. Furthermore, there is no report on the effects of FMeA on DENV1. Interestingly, SOF was reported to inhibit DENV2 RNA replication in human hepatic cells [25]; however, the effects of SOF on DENV1 remain unknown. Thus, in this study, we analyzed the anti-DENV1 effects of SOF and FMeU using the two replicon assays mentioned below.

Although the periodical infections of all four serotypes of dengue have been recorded in Japan and China in the past 25 years [26,27], DENV1 has become the predominant cause of epidemics in 2019 and from 2011 to 2015 in Japan [28]. In addition, DENV1 has become the most prevalent serotype in circulation since 1990 in China [27,29]. Therefore, we focused on DENV1 and established two types of anti-DENV1 drug screening methods, designated as transient replicon assay [30] and replicon-harboring cell assay [31]. In the DNA replicon assay, transient transfection of the luciferase-inserted DENV1 replicon-expression plasmid DNA (DGL2) into an optional cell line is required. In the replicon-harboring cell assay, the luciferase-inserted DENV1 RNA replicon–stably transfected human Huh7 cell is used. These methods have provided novel anti-DENV compounds, including the following DENV RdRp inhibitors: bromocriptine (dopamine agonist) [31], hirsutine (indole alkaloid of *Uncaria rhynchophylla*) [32], stearoyl-CoA desaturase-1 (SCD1) [33], and the imidazole nucleoside derivatives, such as the 4′-thio derivatives of EICAR (5-ethynyl-(1-β-D-ribofuranosyl) imidazole-4-carboxamide) [34]. 

In the transient replicon assay [30], an optional cell line is transiently transfected with the DENV1 replicon–expression plasmid DNA (DGL2), in which the genes encoding the structural proteins (C, E, and preM) are substituted by the secretory *Gaussia* luciferase. When the test compound is evaluated, transfected cells are cultured for 24 h in media containing the test compound. In the transfected cells, the DGL2 plasmid is transcribed into the (+)-stranded DENV1 RNA replicon by cellular RNA polymerase II. This (+)-stranded DENV1 RNA will be used as a template for the translation of *Gaussia* luciferase and the viral enzymes (NS1, NS2A, NS2B, NS3, NS4A, NS4B, and NS5/RdRp). The NS5/RdRp consecutively replicates the (+)- and (−)-stranded RNA replicon from the (−)- and (+)-stranded replicon, and the (+)-stranded replicon is translated into viral enzymes and the luciferase [30]. Importantly, the secreted luciferase activity in the culture supernatant is proportional to the amount of RNA replicon replicated by the RdRp [30]. Thus, the inhibitory effect of the test compound on DENV1 replicon replication can be evaluated by measuring luciferase activity in the culture supernatant of DNA replicon-transfected cells treated with the test compound. In the replicon-harboring cell assay, we used a human hepatoma Huh7 cell line stably transfected with the DENV1 subgenomic RNA replicon in which the C-, E-, and preM-coding regions were substituted with *Gaussia* luciferase-coding RNA [31]. Since human hepatic cells express abundant metabolic enzymes for nucleic acid, human hepatoma Huh7 was used for replicon-harboring cells. Similar to the transient replicon assay, the replication of the RNA replicon can be evaluated by measuring luciferase activity in the culture supernatant of RNA replicon-harboring Huh7 cells. We attempted the transfection with DENV1 replicon into not only Huh7 but also HepG2 cells. However, it was hard to establish a stable HepG2 cell line due to the low transfection efficiency. 

Here, we evaluated the effects of SOF and FMeU on DENV1 replication using two types of assays mentioned above. Moreover, we analyzed the effects of SOF and FMeU on infectious DENV1 production by conducting cytopathic effect (CPE)-based plaque assays of DENV1-infected cells. These assays showed that SOF exerted inhibitory effects on DENV1 genome replication in human hepatic Huh7 cells but not in hamster BHK-21 cells. In contrast, FMeU did not suppress DENV1 replication in those cell lines. 

## 2. Results

### 2.1. Replication of the DENV1 Replicon in Human Hepatic Huh7 Cells Is Suppressed by SOF but Not by FMeU

**Figure 1:**
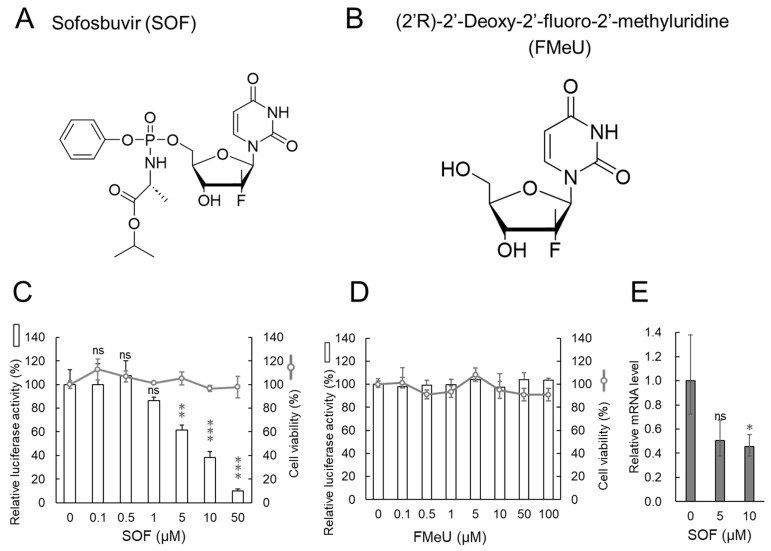
The replicon-harboring cell assay revealed that the replication of the DENV1 RNA replicon was decreased by SOF but not by FMeU: (**A**,**B**) structures of SOF and FMeU. (**C**,**D**) The effects of SOF and FMeU on DENV1 RNA replicon replication using the replicon-harboring cell assay. DENV1 RNA replicon-harboring Huh7 cells were treated with SOF (**C**) or FMeU (**D**) for 48 h, and the culture media were harvested. Next, the Gaussia luciferase activities in the culture media were quantitated. In addition, the cell viabilities of replicon-harboring Huh7 cells treated with SOF (**C**) or FMeU (**D**) for 48 h were measured. The luciferase activity (bar graph) and the cell viability (line graph) in DMSO-treated cells were defined as 100%. (**E**) The effects of SOF on the replication of the DENV1 RNA replicon assessed by RT-qPCR. The replicon-harboring Huh7 cells were treated with SOF for 48 h, and the amount of DENV1 RNA replicon was measured by RT-qPCR. The DENV1 RNA replicon levels were normalized to the GAPDH mRNA levels. The amount of DENV1 replicon in DMSO-treated cells was defined as 1.0. * *p* < 0.05, ** *p* < 0.01, and *** *p* < 0.001 indicate statistically significant differences compared with DMSO-treated cells. ns, not significant.

The structures of SOF and FMeU are shown in Figure 1A,B, respectively. First, we asked whether SOF or FMeU inhibited the replication of the DENV1 replicon using the RNA replicon cell assay, in which the human hepatic Huh7 cells stably harbor the DENV1 RNA replicon (bar graphs in Figure 1C,D). The cytotoxic effects of SOF and FMeU on DENV1 replicon-harboring Huh7 cells were also examined with a cell viability assay (line graphs in Figure 1C,D). Our data showed that 5–50 µM of SOF, which did not affect the cell viability of replicon-harboring Huh7 cells, significantly inhibited the replication of the DENV1 RNA replicon in a dose-dependent manner (Figure 1C). Meanwhile, FMeU (even at a high dose) did not inhibit the replication of the DENV1 replicon (Figure 1D). In particular, a comparison of 50 µM of SOF with 0 µM of SOF revealed that SOF decreased the replication of the DENV1 replicon by approximately 90% without any cytotoxicity. The IC_50_, CC_50_, and selective index (S.I.) values of tested drugs in the stable RNA replicon cell assay are summarized in Table 1. It has been previously reported that ribavirin inhibits the genome replication of flaviviruses, including DENV [35,36,37]. Therefore, ribavirin was used as a control to compare the inhibitory activity. The S.I. of SOF was estimated as >12.0, which was close to the S.I. of ribavirin (>17.4). In addition, the decrease in DENV1 RNA replicon within SOF-treated replicon-harboring Huh7 cells was also confirmed by direct measuring of DENV1 RNA using RT real-time PCR (Figure 1E). Thus, the result of the replicon-harboring cell assay was supported by the PCR-based quantitation data.

### 2.2. SOF Suppresses the Replication of the DENV1 Replicon in Human Huh7 Cells but Not in Hamster BHK-21 Cells

**Figure 2:**
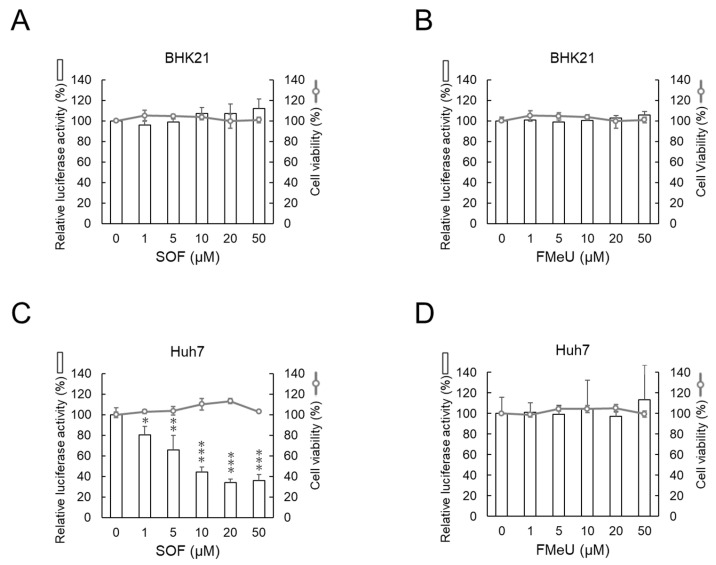
The transient replicon assay showed that replication of the DENV1 replicon was decreased by SOF in human Huh7 cells but not in hamster kidney BHK-21 cells. Evaluation of the effects of SOF or FMeU on replication of the DENV1 DNA replicon in baby hamster kidney fibroblast BHK-21 cells (**A**,**B**) or human hepatic Huh7 cells (**C**,**D**) using the transient replicon assay. Transiently, DENV1 replicon expression plasmid-transfected hamster kidney BHK-21 cells (**A**,**B**) or human hepatic Huh7 cells (**C**,**D**) were treated with SOF (**A**,**C**) or FMeU (**B**,**D**) for 48 h. Drug-treated cells were further cultured for 24 h in fresh media containing 2% FBS and the test drug. The culture media were harvested, and the Gaussia luciferase activities in the media were measured. Cell viabilities of BHK-21 cells (**A**,**B**) or Huh7 cells (**C**,**D**) treated with SOF (**A**,**C**) or FMeU (**B**,**D**) for 72 h were also measured. (**A**–**D**) The luciferase activity (bar graph) and the cell viability (line graph) in DMSO-treated cells were defined as 100%. * *p* < 0.05, ** *p* < 0.01, and *** *p* < 0.001 indicate statistically significant differences compared with DMSO-treated cells.

The replicon-harboring cell assay revealed that the replication of the DENV1 RNA replicon in human hepatic Huh7 cells was suppressed by SOF but not by FMeU. Next, we evaluated the effects of SOF and FMeU using the transient replicon assay, which requires transient transfection with the DENV1 replicon expression plasmid into optional cells. Baby hamster kidney fibroblast BHK-21 and human hepatic Huh7 cells were used as the test cells and were transiently transfected with the DENV1 plasmid, and the effects of SOF and FMeU on DENV1 replicon replication were evaluated. Neither SOF nor FMeU suppressed the DENV1 replicon replication in hamster BHK-21 cells (bar graphs in Figure 2A,B), whereas the DENV1 DNA replicon replication in human Huh7 cells was decreased to approximately 40% by SOF (10–50 µM), compared with that of 0 µM of SOF (Figure 2C). In contrast to SOF, FMeU had no effect on the DENV1 DNA replicon replication in both human Huh7 and hamster BHK-21 cells (Figure 2B,D). The cytotoxic effects of SOF or FMeU on BHK-21 and Huh7 cells were examined and showed that the concentration range of SOF or FMeU used in this study did not affect cell viability (line graphs in Figure 2A–D). The IC50 and S.I. of tested drugs in the transient replicon assay are shown in Table 2. Taken together, these results indicate that SOF suppresses DENV1 replicon replication in human Huh7 cells but not in hamster BHK-21 cells, while the replication of the DENV1 replicon in both BHK-21 and Huh7 cells is insensitive to FMeU. 

### 2.3. SOF Suppresses DENV1 Production in Human Hepatic Huh7 Cells but Not in Monkey Kidney Vero Cells

**Figure 3:**
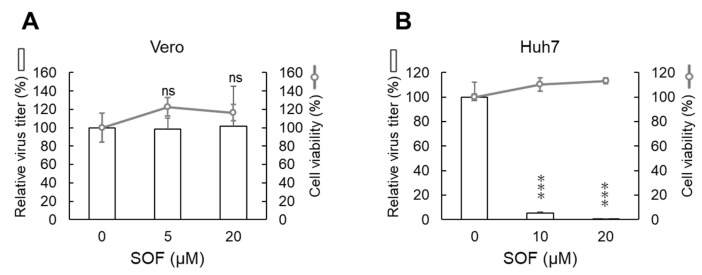
SOF suppressed DENV1 production in Huh7 cells but not in Vero cells: (**A**,**B**) Evaluation of the effects of SOF on the infectious DENV1 production in monkey kidney Vero and human hepatic Huh7 cells. Vero or Huh7 cells were infected with DENV1 (02-20 strain) for 1 h, and the cells were cultured with fresh media containing SOF for 3 days. The virus amount in the harvested cultured supernatant was assessed by a CPE-based plaque assay using Vero cells. The virus amount (bar graph) and the cell viability (line graph) in DMSO-treated cells were defined as 100%. *** *p* < 0.001 indicates statistically significant differences compared with DMSO-treated cells. ns, not significant.

The transient replicon assay demonstrated that SOF suppressed the replication of the DENV1 replicon in human hepatic Huh7 cells but not in baby hamster kidney fibroblast BHK-21 cells. In order to gain further evidence on the SOF-mediated suppression of DENV genome replication, we evaluated the effects of SOF in DENV1-infected African green monkey Vero cells (Figure 3A) and human Huh7 cells (Figure 3B). Vero or Huh7 cells were infected with DENV1 (02-20 strain) for 1 h, and the culture media were replaced with fresh media containing different concentrations of SOF. The cells were cultured for 3 days, and the culture supernatants were harvested. Viral titers in the culture supernatants were quantitated using a CPE-based plaque assay. We found that 10 and 20 µM of SOF inhibited DENV1 production by over 90% in human Huh7 cells; however, these drastic inhibitory effects were not detected in monkey Vero cells. The IC50 and S.I. of SOF are shown in Table 3. Interestingly, unlike human Huh7 cells, SOF does not suppress DENV1 replication in both baby hamster BHK-21 cells (Figure 2A) and African green monkey Vero cells (Figure 3A).

## 3. Discussion

Our study revealed that the replication of the DENV1 replicon in human hepatic Huh7 cells was decreased by SOF but not by FMeU (Figure 1). The transient replicon assay also showed that replication of the DENV1 replicon was suppressed by SOF in human hepatic Huh7 cells but not in baby hamster kidney fibroblast BHK-21 cells (Figure 2). Moreover, replication of the DENV1 replicon in both human Huh7 cells and hamster BHK-21 cells was not affected by FMeU (Figure 2). An inhibition assay using DENV1-infected cells showed that SOF suppressed viral production in human hepatic Huh7 cells but not in monkey kidney Vero cells (Figure 3). To our knowledge, this is the first report demonstrating that SOF suppresses DENV1 replication in human hepatic cells.

**Figure 4:**
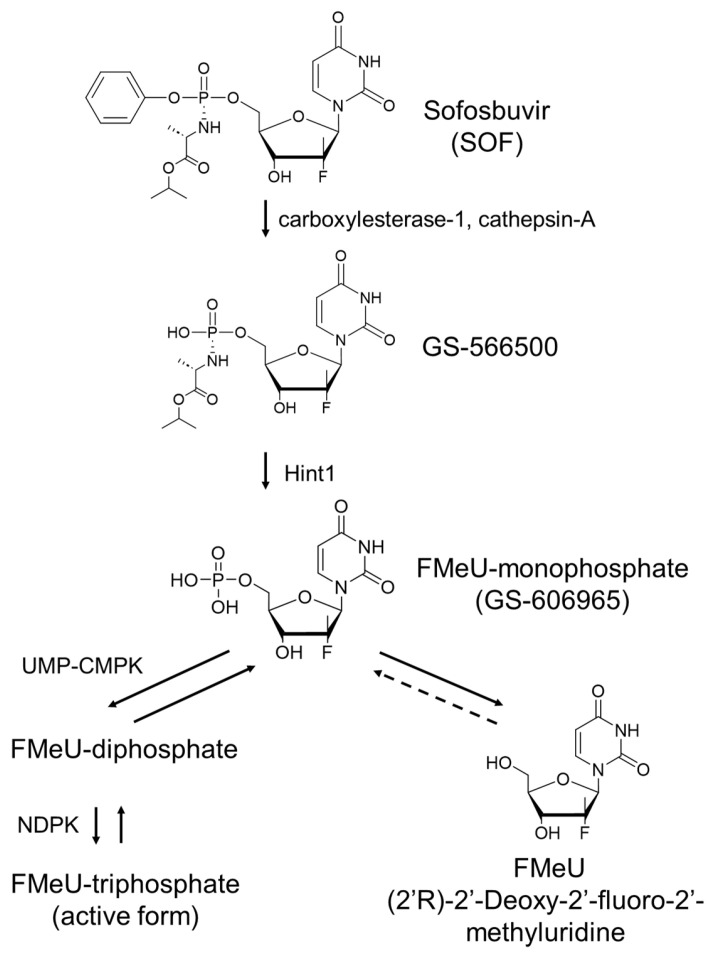
Predictable metabolic pathway of SOF in human hepatocyte. SOF is converted into GS-566500 by carboxylesterase-1 and cathepsin-A, and subsequently, GS-566500 is converted into FMeU-monophosphate (GS-606965) by histidine triad nucleotide-binding protein-1 (Hint1). FMeU-monophosphate (FMeU-MP) is metabolized into FMeU-triphosphate (FMeU-TP) by nucleotide kinases or into FMeU by dephosphorylation. UMP-CMPK, uridine monophosphate/cytidine monophosphate kinase; NDPK, nucleoside diphosphate kinase.

Several nucleoside and nucleotide analogs have been examined as inhibitors of the DENV RdRp (NS5) [12,14]. The first step in the nucleoside analog-mediated DENV RdRp inhibition pathway is the 5’-monophosphorylation of the nucleoside analog by cellular nucleoside kinases. Next, these nucleoside 5’-monophosphates further require diphosphorylation and subsequent triphosphorylation by cellular nucleotide kinases in order to become active forms. The resulting nucleoside 5’-triphosphates can inhibit the DENV RdRp, which in turn inhibits the synthesis of DENV RNA [16,17]. Figure 4 shows the SOF metabolic pathway that is proposed by the manufacturing company, Gilead Sciences [24]. In the human hepatic cell, SOF is rapidly converted into the first SOF-metabolite, GS-566500, by carboxylesterase-1- and cathepsin-A-mediated detachment of iso-propanol and phenol. Subsequently, GS-566500 is converted into FMeU-monophosphate (FMeU-MP/GS-606965) by the histidine triad nucleotide-binding protein 1 (HINT1)-mediated detachment of alanine. FMeU-MP is metabolized into the active form FMeU-triphosphate (FMeU-TP) through di- and triphosphorylation by nucleotide kinases (UMP-CMPK and NDPK) or into FMeU by dephosphorylation. When SOF is administered to adult humans, over 90% of SOF is present as FMeU in the blood [24]. Our data showed that SOF only suppresses DENV1 replicon replication in human hepatic cells, and FMeU does not suppress DENV1 replicon replication in animal cells and human hepatic cells. Therefore, we hypothesize that FMeU is not metabolized into FMeU-MP by 5’-phosphorylation because FMeU cannot be a substrate of the nucleoside kinase of animal cells, including humans. Meanwhile, our results strongly suggest that SOF is metabolized into FMeU-MP and FMeU-TP by carboxylesterase-1, Hint1, and nucleotide kinases in human hepatic cells but not in other animal cell types. Xu et al. also showed that SOF inhibited the replication of a DENV2 RNA replicon in human hepatic Huh7 cells, and FMeU-TP decreased the polymerase activity of the DENV2 RdRp in vitro [25]. Other possible mechanisms for the cell-type-specific effects may be that the substrate specificities or the expression levels of SOF metabolic enzymes, such as carboxylesterase-1, cathepsin-A, HINT1, and nucleotide kinases, may be different among hamster kidney BHK-21, monkey kidney Vero, and human hepatic Huh7 cells. Concerning the target cells, DENV can replicate in various cells and organs, including the liver, brain, kidney, spleen, lung, and lymph nodes [1,2]. However, DENV infects dendritic cells, monocytes, and macrophages in the first infection by a DENV-infected Aedes mosquito [1,2]. Thus, we need to evaluate an anti-DENV activity in cell lines derived from these organs other than hepatic Huh7. Moreover, it is an important issue to compare the expression levels of SOF metabolic enzymes among these organs, and these issues should be addressed in the future.

**Figure 5:**
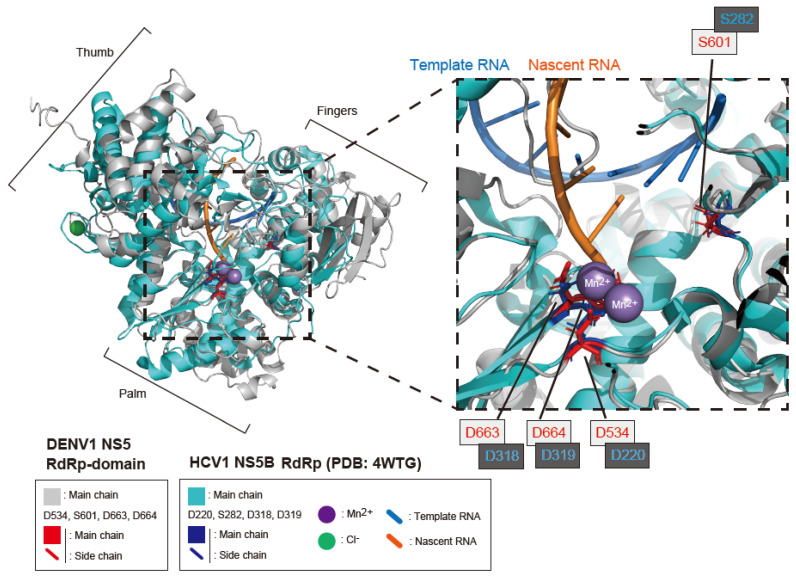
The DENV1 RdRp prediction model and its comparison with the HCV RdRp. (**Left panel**) comparison between the AI-predicted DENV1 RdRp and the X-ray crystal structure of the HCV NS5B/RdRp. The structure of the DENV1 RdRp domain (a.a. 273–900) was predicted by AlphaFold 2. The main chain of the DENV1 RdRp is shown in gray. In the DENV1 RdRp, the catalytic residues (D534, D663, and D664) as well as S601 (which is analogous to S600 of the DENV2 RdRp and speculated to be involved in SOF incorporation) are shown by red sticks. The predicted DENV1 RdRp was superimposed on the HCV NS5B/RdRp complex (elucidated by X-ray crystal structure analysis) containing template RNA, nascent RNA, SOF metabolite, Mn^2+^ ions, and a Cl^−^ ion (PDB: 4WTG). The main chain of the HCV NS5B/RdRp is shown in light blue, and the catalytic residues (D220, D318, and D319) as well as S282 (which is analogous to S601 of DENV1) are shown by dark blue sticks. The Mn^2+^ and Cl^−^ ions are shown in purple and green, respectively. The template RNA is shown in a blue bold line, and the nascent RNA is shown in an orange bold line. (**Right panel**) the enclosed catalytic core region of the DENV1 and HCV RdRps.

To examine the structural differences between the RdRps of DENV1 and HCV, their conformations were compared (Figure 5). The structure of the DENV1 strain 02-20 RdRp (a.a. 273–900) was estimated by AlphaFold 2 and visualized with the open-source software PyMOL (ver 2.5.0). The structure of the HCV NS5B RdRp, along with SOF metabolite (FMeU- diphosphate), nascent RNA (orange stick), template RNA (blue stick), Mn^2+^ ions, and a Cl^−^ ion, was elucidated by X-ray crystal structure analysis [22], and the structural data (PDB; 4WTG) of the HCV RdRp-RNA-ions complex were visualized by PyMOL. The predicted structure of the DENV1 RdRp showed the canonical cupped right-handed configuration conserved among flaviviral RdRps. Each domain structure of the DENV1 RdRp (thumb, palm, and fingers) does not closely match with the domains of the HCV RdRp (Figure 5 left panel), which is similar to the DENV3 RdRp [38]. However, the amino acid configuration of the DENV1 catalytic core region is in good agreement with the HCV RdRp (Figure 5 right panel) and the DENV3 RdRp [38]. The catalytic residues (D534, D663, and D664) and an important residue S601 (involved in the incorporation of SOF) of the DENV1 RdRp are shown as red sticks. The comparable residues of HCV are shown as dark blue sticks. The purpose of these structural comparisons of the HCV and DENV1 RdRps is to expand the use of SOF to treat DENV1 infection. Wu et. al. demonstrated the structure of the elongation complex of DENV2 RdRp and the refolding mechanism of the priming element (PE) in the thumb domain of DENV2 RdRp by X-ray crystallography [39]. DENV2 RdRp utilizes a PE in its thumb domain to facilitate the initiation of RNA synthesis. At the transition from initiation to elongation in RNA synthesis, the PE in the thumb domain of DENV2 RdRp was reported to demonstrate dramatic refolding to make space for the template–nascent RNA duplex. The PE of DENV1 RdRp is located in the right upper side of its thumb domain, close to the left upper side of its fingers domain. 

**Figure 6:**
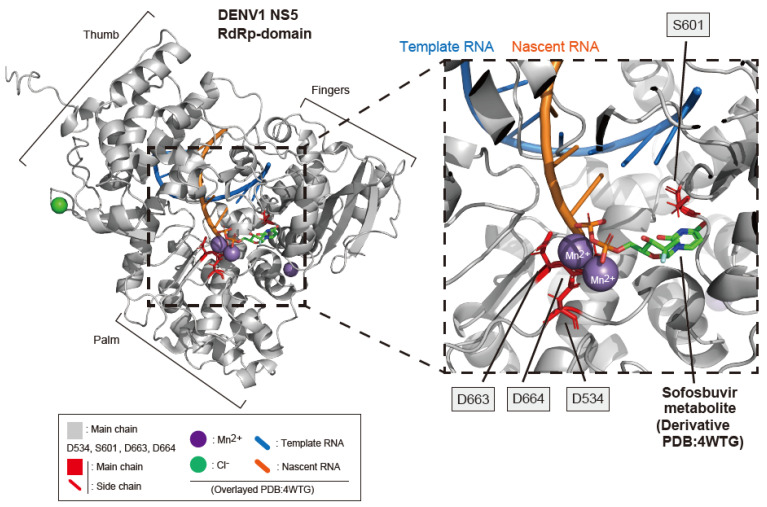
The docking structure of the DENV1 RdRp catalytic site and phosphorylated SOF. (**Left panel**) the docking model of the DENV1 RdRp domain (a.a. 273–900) and SOF metabolite. The main chain of the DENV1 RdRp is shown in gray. The catalytic residues (D534, D663, and D664) as well as S601 are shown as red sticks. The SOF metabolite (FMeU-diphosphate) is shown as a stick model marked with each atom. The template RNA (blue bold line), nascent RNA (orange bold line), Mn^2+^ ions (purple), and a Cl^−^ ion (green), which were obtained from the structural data of the HCV NS5B/RdRp-RNA-SOF metabolite–ions complex (PDB: 4WTG), were superimposed on the docking model of the SOF-DENV1 RdRp. (**Right panel**) the enlarged region within the dotted line in the left panel.

In order to gain further insight into the substrate recognition of the DENV1 RdRp, the docking structure between its catalytic site and the SOF metabolite (FMeU-diphosphate) was visualized by AlphaFold 2, UCSF Chimera, and AutoDock Vina. The binding structure of the SOF metabolite and the DENV1 RdRp (a.a. 273–900) is shown in Figure 6. Template RNA (blue stick), nascent RNA (orange stick), Mn^2+^ ions, and a Cl^−^ ion were superimposed from the structural data of the HCV RdRp-template (and nascent) RNA-SOF metabolite-Mn^2+^-Cl^−^ complex (PDB: 4WTG) [22]. The docking model showed that the phosphorylated SOF fits well into the DENV1 RdRp catalytic site (consisting of two Mn^2+^ ions and the catalytic residues (D534, D663, and D664)) via its phosphate groups (Figure 6 right panel). The catalytic residues (D534, D663, and D664) of the DENV1 RdRp are analogous to D533, D663, and D664 of DENV2 RdRp [16], as well as D220, D318, and D319 of HCV RdRp/NS5B [22]. Additionally, the uracil base of the SOF metabolite associates with S601 in the DENV1 RdRp, which is analogous to S600 of DENV2 RdRp and S282 of HCV RdRp/NS5B (Figure 6 right panel). The S600 residue of DENV2 RdRp is related to the resistance to nucleoside analog inhibitors and is important for SOF incorporation into DENV2 RdRp [16,25]. The docking state of the SOF metabolite to DENV1 RdRp did not completely agree with that of HCV RdRp/NS5, but it did resemble the direction and coordinate location in the catalytic site.

There are four serotypes of DENV, and past infection by different types is closely related to developing dengue fever. Recovery from infection by a certain serotype provides long immunity against that particular serotype. However, cross-protection to the other serotypes after recovery is temporary and restricted. The subsequent heterotypic infection increases the risk of developing severe dengue fever. In fact, multiple dengue serotypes circulate in hyperendemic regions, which creates critical importance for the development of an antiviral drug and a vaccine against all four serotypes. Since SOF is active for DENV1 (this study) and DENV2 [25], it can be expected that SOF is a pan-serotype inhibitor targeting DENV1-4 RdRps. An amino acid sequence alignment of DENV1-4 RdRps (NS5s) and HCV RdRp (NS5B) is depicted in Supplementary Figure S1. This analysis shows that the amino acid sequence of DENV1 RdRp has a high homology to those of DENV2-4 RdRps. Moreover, the amino acid sequence of DENV1 RdRp has partial homology to that of HCV RdRp. In particular, the catalytic residues (D534, D663, and D664) of DENV1 RdRp and S601 (a binding amino acid to the uracil in SOF) of DENV1 RdRp are highly conserved among DENV1-4 RdRps and HCV RdRp. This information also supports that SOF is a pan-serotype DENV inhibitor, and SOF is active for not only HCV but also DENV.

Evidence showing that SOF has anti-DENV1 activity without leading to lethality in an animal model is important for advancing this compound into future clinical studies. Although the evaluation of the anti-DENV activity of SOF in an animal model is required, we currently do not have a suitable animal model for drug screening against DENV infection. This issue should be addressed in future studies. In summary, we have demonstrated that SOF has the ability to suppress the replication of DENV1 in human hepatic cells, whereas FMeU, the nucleoside form of SOF, does not. These findings suggest that SOF could serve as a novel therapeutic agent against DENV1 infection.

## 4. Material and Methods

### 4.1. Cells and Compounds

Human hepatoma cells (Huh-7, RCB1366), baby hamster kidney fibroblast cells (BHK-21, RCB1423), and African green monkey kidney cells (Vero, RCB0001) were provided by the RIKEN BioResource Research Center (https://web.brc.riken.jp/ja/ (accessed on 10 December 2023)). Huh7 and Vero cells were cultured in DMEM (Wako, Tokyo, Japan), supplemented with 10% fetal bovine serum (FBS). BHK-21 cells were cultured in E-MEM (Wako) with 10% FBS. Ribavirin and (2′R)-2′-deoxy-2′-fluoro-2′-methyluridine (FMeU) were purchased from Tokyo Chemical Industry (Tokyo, Japan), and sofosbuvir (SOF) was obtained from ChemScene LLC (Monmouth Junction, NJ, USA)

### 4.2. Cell Viability Assay

The cells were maintained in E-MEM or DMEM with 10% FBS. The cells were then seeded in 96-well plates at 5 × 10^3^ cells/well in 100 µL of media with or without the compound at various concentrations. Next, the cells were incubated for 48 or 72 h. The number of viable cells was estimated using the Cell Count Reagent SF (Nacalai Tesque, Kyoto, Japan) [40]. The optical density at 450 nm (reference wavelength: 620 nm) was measured using a Tecan M200 microplate spectrophotometer (Tecan, Kanagawa, Japan) and was expressed as a percentage of the value of untreated cells (defined as 100%). The 50% cytotoxic concentration (CC_50_) was defined as the compound concentration at which the cell viability decreased by 50% compared with the control.

### 4.3. Replicon-Harboring Cell Assay

The establishment of a stable RNA replicon cell assay was described previously [31]. Briefly, the DENV1 RNA replicon was synthesized by in vitro transcription using the DENV1 plasmid DNA (DGL2) as a template. In the DGL2 construct, the genes encoding the structural proteins of DENV1 (C, E, and preM) are substituted with the secretory Gaussia luciferase and Neomycin-resistant gene. The synthesized RNA replicon was transfected into Huh7 cells, and the transformed cells were selected and maintained with media containing G418. The Huh7 cells stably harboring the replicon DENV1 RNA were seeded in 96-well plates at 1 × 10^4^ cells/well in 0.2 mL of 10% FBS-containing DMEM. The cells were then cultured for 12 h. The media were replaced with 0.2 mL of DMEM supplemented with 2% FBS and the compound, and the cells were incubated for 48 h. Next, the media were replaced with fresh media containing 2% FBS without the compound, and the cells were cultured for an additional 4 h. The Gaussia luciferase activities in the harvested media were detected by a BioLux Gaussia Luciferase Assay kit (New England Biolabs, Ipswich, MA, USA) and were quantitated using a Tecan M200 microplate spectrophotometer. The luciferase activity in DMSO-treated cells was defined as 100%. The 50% inhibitory concentration value (IC_50_) was defined as the compound concentration at which the Gaussia luciferase activity decreased by 50% compared with the DMSO control. The S.I. was obtained by calculating the ratio of the CC_50_ to the IC_50_ (CC_50_/IC_50_) for each compound.

### 4.4. Transient Replicon Assay

A transient replicon assay was conducted using the DENV1 replicon expression plasmid DNA (DGL2) as described previously [30]. Briefly, BHK-21 and Huh7 cells were seeded in 24-well plates at 2 × 10^5^ cells/well in 0.5 mL of 10% FBS-containing E-MEM and DMEM, respectively. The cells were transfected with DGL2 plasmid using X-tremeGENE HP DNA transfection reagent (Roche, Basel, Switzerland). After 6 h, media containing the transfection reagent were replaced with 0.5 mL of medium supplemented with 2% FBS and the compound, and the cells were incubated for 48 h. Next, the media were replaced with fresh media containing 2% FBS and the compound, and the cells were cultured for an additional 24 h. Gaussia luciferase activities in the harvested media were detected with a BioLux Gaussia Luciferase Assay kit (New England Biolabs) and were quantitated using a Tecan M200 microplate spectrophotometer. The luciferase activity in DMSO-treated cells was defined as 100%. 

### 4.5. Quantitative Reverse Transcription PCR (RT-qPCR)

DENV1 RNA replicon-harboring Huh7 cells (3 × 10^5^ cells/well) were seeded in 6-well plates and cultured for 12 h. Test drugs were then added to the media, and the cells were subsequently cultured in the presence (or absence) of the drug for 48 h. mRNA was extracted from the cells using RNAiso Plus (Takara, Osaka, Japan). cDNA was synthesized using a ReverTra Ace qPCR kit (Toyobo, Osaka, Japan) and was subjected to SYBR green real-time PCR [40]. The relative mRNA expression levels were determined by ΔΔCt methods. The primer set for DENV1 NS5 (NS5-forward 5′-AGATGAGCGGTTCTGGGACC-3′ and NS5-reverse 5′-CCTAGCTTTTTCTCTCTCTTCCCCAT-3′) was used to measure the expression level of the DENV1 RNA replicon. The primer set for GAPDH (forward 5’-CATCAAGAAGGTGGTGAAGCAG-3’ and reverse 5’-TGTCGCTGTTGAAGTCAGAGG -3’) was used as an internal control for normalization. For quantification, the expression level of the DENV1 RNA replicon was normalized to the GAPDH mRNA expression level.

### 4.6. DENV1 Infection and CPE-Based Plaque Assay

The 02-20 strain of DENV1 was derived from the full-length infectious cDNA clone, D1 (02-20)/pMW119 [41]. Vero or Huh7 cells were seeded in a 96-well plate (5 × 10^4^ cells/well). At 1 day after seeding, the cells were infected with DENV1 (02-20 strain) at 50 focus forming units per well (or at a multiplicity of infection (MOI) of 1.0.). After 1 h of incubation, the culture supernatant was replaced with media containing E-MEM, 2% FBS, and the test compound. After an additional 3 days of cultivation, the cell culture supernatant was harvested, and the viral titer was assessed by a CPE-based plaque assay.

The CPE-based plaque assay was performed according to a method described below. Vero cells were seeded in 12-well plates (5 × 10^5^ cells/well). Next, the cells were inoculated with harvested culture supernatant for 1 h. Subsequently, E-MEM supplemented with 2% FBS and 1% methylcellulose was added, and the cells were incubated for an additional 6 days. Next, the cells were fixed with 3.7% formaldehyde in PBS and stained with methylene blue. The viral titers were quantitated by counting the number of plaques derived from viral CPEs.

### 4.7. Molecular Docking Modeling

Ligand–protein molecular docking predictions were performed according to Xu T.H. et al. [25] and a helpful handling tutorial [42], with minor modifications. Briefly, the DENV1 strain 02-20 NS5 RdRp domain structure model was predicted by AlphaFold 2.2.0 (https://github.com/deepmind/alphafold (accessed on 15 February 2022)) in its own local environment [43]. The following databases were utilized for AlphaFold 2: Uniclust30 (version 2018_08), MGnify (version 2018_12), PDB70 (downloaded on 15 February 2022), PDB/mmCIF (downloaded on 18 February 2022), and pdb_seqres (downloaded on 18 Ferbuary 2022). The AlphaFold 2 predicted model of the RdRp had a high confidence score (pLDDT > 90) comprising nearly the whole structure, but it did not include the ions, especially Mn^2+^, which is essential for the catalytic function of the RdRp. In order to obtain the coordinate score of the binding ions, the predicted DENV1 RdRp were overlayed and aligned with the HCV NS5B/RdRp–RNA–SOF metabolite–Mn^2+^–ions complex (PDB; 4WTG) [22] by the molecular visualization open-source software, PyMOL (ver 2.5.0). Based on the obtained coordinates, ions were inserted into the predicted structure. On the other hand, the SOF metabolite (FMeU-DP) was extracted from the HCV complex structure (PDB; 4WTG). Using these structures, the docking preparation and calculations were conducted with UCSF chimera (ver 1.16 (built42360)) (https://www.cgl.ucsf.edu/chimera/ (accessed on 15 February 2022)) [44] and AutoDock Vina (ver 1.2.0) (https://vina.scripps.edu (accessed on 15 February 2022)) [45,46], respectively. The docking prediction model of DENV1 RdRp-SOF metabolite was superimposed with RNA strands and Mn^2+^/Cl^−^ ions from the HCV complex structure (PDB; 4WTG) and was visualized with PyMOL.

### 4.8. Statistical Analyses

The statistical significance between each group and the control (DMSO-treated cells) was analyzed by one-way ANOVA followed by Dunnett’s or Tukey’s test for multiple comparisons. Statistically significant data were analyzed with GraphPad prism7 (GraphPad Software, San Diego, CA, USA). * *p* < 0.05, ** *p* < 0.01, and *** *p* < 0.001 indicate statistical significance, and “ns” indicates not significant.

## Figures and Tables

**Table 1:** IC50 and S.I. of SOF, FMeU, and ribavirin calculated using the replicon-harboring cell assay.

Compound	IC_50_ ^a^ (µM)	CC_50_ ^b^ (µM)	S.I. ^c^
SOF	8.31	>100	>12.0
FMeU	>100	>100	-
ribavirin	5.74	>100	>17.4

^a^: Inhibitory concentration of the compound that reduces Gaussia luciferase activity by 50%. ^b^: Cytotoxic concentration of the compound that reduces cell viability by 50%. ^c^: Selective index (CC_50_/IC_50_).

**Table 2:** IC50 and S.I. of SOF and FMeU calculated using the transient replicon assay.

Cell	Compound	IC_50_ ^a^ (µM)	CC_50_ ^b^ (µM)	S.I. ^c^
BHK-21	SOF	>100	>100	-
	FMeU	>100	>100	-
Huh7	SOF	8.34	>100	>12.0
	FMeU	>100	>100	-

^a^: Inhibitory concentration of the compound that reduces Gaussia luciferase activity by 50%. ^b^: Cytotoxic concentration of the compound that reduces cell viability by 50%. ^c^: Selective index (CC_50_/IC_50_).

**Table 3:** IC_50_ and S.I. of SOF evaluated by infectious DENV1 and CPE-based plaque assay.

Cell	Compound	IC_50_ ^a^ (µM)	CC_50_ ^b^ (µM)	S.I. ^c^
Vero	SOF	>20	>50	-
Huh7	SOF	5.24	>50	>9.54

^a^: Inhibitory concentration of the compound that reduces Gaussia luciferase activity by 50%. ^b^: Cytotoxic concentration of the compound that reduces cell viability by 50%. ^c^: Selective index (CC_50_/IC_50_).

## Data Availability

Underlying data and the accession numbers are available in the main text. All other raw data will be shared upon request.

## Supplementary Materials

The supporting information can be downloaded at https://www.mdpi.com/article/10.3390/ijms25042022/s1.
