# Supplementary material for: Sofosbuvir Suppresses the Genome Replication of DENV1 in Human Hepatic Huh7 Cells
Source: Int J Mol Sci. 2024 Feb 7;25(4):2022. doi: 10.3390/ijms25042022 (PMC10889370; doi:10.3390/ijms25042022)
Supplement: Supplementary file 1 [file ijms-25-02022-s001.zip › ijms-2822101-supplementary.pdf]

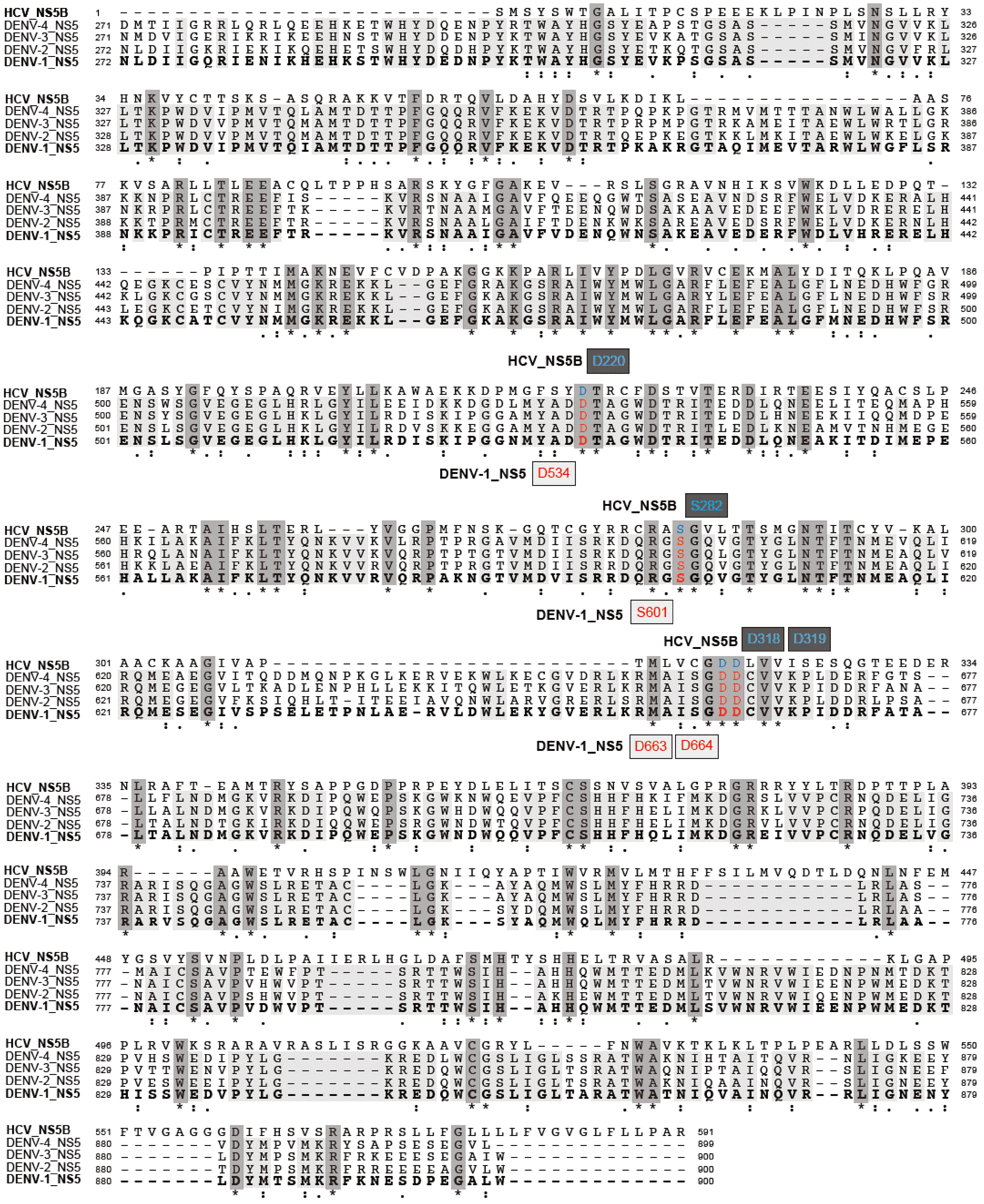

Supplementary Figure S1. Amino-acid sequence alignment of DENV1-4 RdRp (NS5) and HCV RdRp (NS5B).

Amino acid sequences of DENV RdRp (NS5) and HCV RdRp (NS5B) were obtained from the NCBI database [HCV isolate JFH-1 (GenBank: AB047639.1), DENV1 strain NIID02-20 (GenBank: AB178040), DENV2 strain Thailand/16681/1984 (GenBank: M84727.1), DENV3 strain Sri Lanka/1266/2000 (GenBank: AY099336.1), and DENV4 strain Dominica/814669/1981 (GenBank: QHB20503)]. The raw data used for the alignment were obtained with the Clustal Omega program (EMBL-EBI; https://www.ebi.ac.uk/Tools/msa/clustalo/). The completely matched amino-acid residues among HCV and DENV1-4s are shown in black letters with a dark-gray background. The completely matched amino-acid residues among DENV1-4s are shown in black letters with a light-gray background. An asterisk (*) indicates positions which have a single, fully conserved residue. A colon (:) indicates conservation between groups of strongly similar properties. A period (.) indicates conservation between groups of weakly similar properties.
